# Evaluation of Mesenchymal Stem Cell Sheets Overexpressing BMP-7 in Canine Critical-Sized Bone Defects

**DOI:** 10.3390/ijms19072073

**Published:** 2018-07-17

**Authors:** Yongsun Kim, Byung-Jae Kang, Wan Hee Kim, Hui-suk Yun, Oh-kyeong Kweon

**Affiliations:** 1 BK21 PLUS Program for Creative Veterinary Science Research, Research Institute for Veterinary Science and College of Veterinary Medicine, Seoul National University, Seoul 08826, Korea; chris1st@snu.ac.kr (Y.K.); whkim@snu.ac.kr (W.H.K.); 2 College of Veterinary Medicine and Institute of Veterinary Science, Kangwon National University, Chuncheon 24341, Korea; bjkang@kangwon.ac.kr; 3 Powder and Ceramics Division, Korea Institute of Materials Science, Changwon 51508, Korea; yuni@kims.re.kr

**Keywords:** bone regeneration, bone morphogenetic protein 7, demineralized bone matrix, cell sheets, mesenchymal stem cells

## Abstract

The aim of this study was to investigate the in vitro osteogenic capacity of bone morphogenetic protein 7 (BMP-7) overexpressing adipose-derived (Ad-) mesenchymal stem cells (MSCs) sheets (BMP-7-CS). In addition, BMP-7-CS were transplanted into critical-sized bone defects and osteogenesis was assessed. BMP-7 gene expressing lentivirus particles were transduced into Ad-MSCs. BMP-7, at the mRNA and protein level, was up-regulated in BMP-7-MSCs compared to expression in Ad-MSCs. Osteogenic and vascular-related gene expressions were up-regulated in BMP-7-CS compared to Ad-MSCs and Ad-MSC sheets. In a segmental bone-defect model, newly formed bone and neovascularization were enhanced with BMP-7-CS, or with a combination of BMP-7-CS and demineralized bone matrix (DBM), compared to those in control groups. These results demonstrate that lentiviral-mediated gene transfer of BMP-7 into Ad-MSCs allows for stable BMP-7 production. BMP-7-CS displayed higher osteogenic capacity than Ad-MSCs and Ad-MSC sheets. In addition, BMP-7-CS combined with demineralized bone matrix (DBM) stimulated new bone and blood vessel formation in a canine critical-sized bone defect. The BMP-7-CS not only provides BMP-7 producing MSCs but also produce osteogenic and vascular trophic factors. Thus, BMP-7-CS and DBM have therapeutic potential for the treatment of critical-sized bone defects and could be used to further enhance clinical outcomes during bone-defect treatment.

## 1. Introduction

Trauma, tumor resection, and skeletal abnormalities are the main reasons for large bone defects. Successful repairs of bone-defect injuries are a major issue in reconstructive surgery. Autologous bone tissue transplantation is considered the most appropriate technique to treat large bone defects; however, this method has several drawbacks, including donor site morbidity, chronic pain, and inadequate volume of graft material [1].

To preclude these problems, bone tissue engineering using stem cells has been developed as an alternative strategy of bone graft. In particular, mesenchymal stem cells (MSCs) show great potential for therapeutic use in bone tissue engineering due to their capacity for osteogenic differentiation and regeneration [2]. Cell sheets are beneficial for cell transplantation because cell-cell junctions and endogenous extracellular matrix (ECM) are preserved, thereby ensuring homeostasis of the cellular microenvironment for the delivery of growth factors and cytokines that promote tissue repair over a prolonged period of time [3,4]. 

Osteoblast differentiation from multipotent stem cells is regulated by bone morphogenetic proteins (BMPs), which are members of the transforming growth factor (TGF) superfamily. Many studies have demonstrated that recombinant BMPs can stimulate bone formation [5,6,7]. However, large doses of recombinant BMP are required to produce an adequate biological response, which can be costly. The development of gene-modified tissue engineering is an attractive approach with great potential for repairing bone defects. Lentiviral-based gene therapy systems offer prolonged gene expression, and might be ideal for the treatment of large bone defects that require a long-term osteoinductive stimulus or in cases wherein the host biological environment has been compromised [8,9].

Among bone substitutes, demineralized bone matrix (DBM) is effective and widely used clinically. DBM is prepared by acid extraction from allograft bone. This process exposes BMPs and other peptide-signaling molecules on the retained collagen skeleton to improve the osteoinductive and osteoconductive potential [10]. Growth factors such as insulin-like growth factor-1 and TGF-β have also been identified in DBM [11]. DBM has no immunological rejection as the antigenic surface structure of the bone is destroyed during demineralization by acid [12]. Therefore, the DBM has become a suitable alternative to autologous bone grafts in certain clinical situations such as bone defects and comminuted fracture with bone loss [13,14].

Osteogenesis, osteoinduction, and osteoconduction are considered important for bone regeneration; therefore, treatment strategies should include all prerequisites of optimal bone healing, such as osteogenic cells, osteoinductive factors, and osteoconductive matrix. We hypothesized that combining MSC sheets and DBM could accelerate and enhance bone regeneration in large bone defects. In this study, canine adipose-derived MSCs (Ad-MSCs) overexpressing BMP-7 were produced and their cell sheets were generated. The osteogenic potential of Ad-MSC sheets and BMP-7 overexpressing Ad-MSC sheets (BMP-7-CS) was investigated in vitro. In addition, BMP-7-CSs with or without DBM particles, were constructed and assessed for their in vivo osteogenic potential after transplantation into critical-sized bone defects in dogs.

## 2. Results

### 2.1. Gene Transduction and BMP-7 Secretion In Vitro

**Figure 1:**
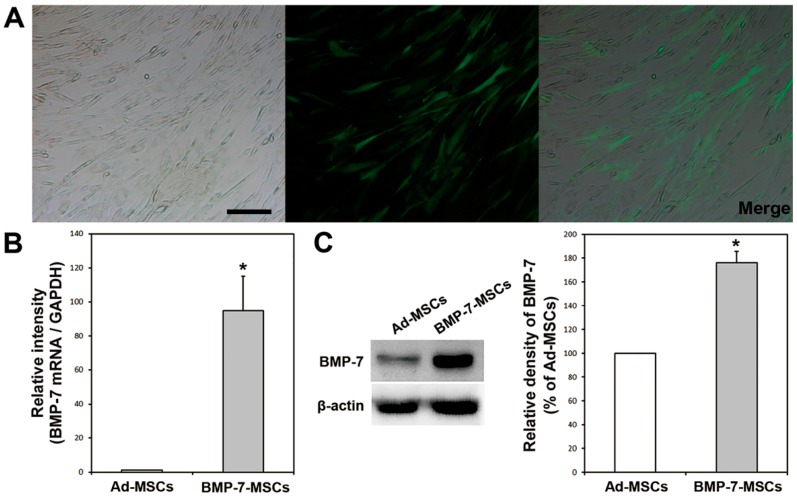
(**A**) Green fluorescence protein (GFP) was observed in adipose-derived mesenchymal stem cells (Ad-MSCs) after lentivirus transduction; (**B**) *Bone morphogenetic protein 7 (BMP-7)* mRNA levels in BMP-7 overexpressing Ad-MSCs (BMP-7-MSCs) were up-regulated compared to that of Ad-MSCs (* *p* < 0.05); (**C**) BMP-7 protein expression in BMP-7-MSCs was higher than in Ad-MSCs (* *p* < 0.05). Bar: 50 μm.

Canine Ad-MSCs were transduced with lentiviral vector encoding the *green fluorescent protein (GFP)* and *BMP-7* gene. GFP-expressing BMP-7 overexpressing Ad-MSCs (BMP-7-MSCs) were identified after transduction. The expression of *BMP-7* mRNA and BMP-7 protein were significantly upregulated in BMP-7-MSCs compared to that of Ad-MSCs (*p* < 0.05, Figure 1).

### 2.2. Alkaline Phosphatase (ALP) Activity

**Figure 2:**
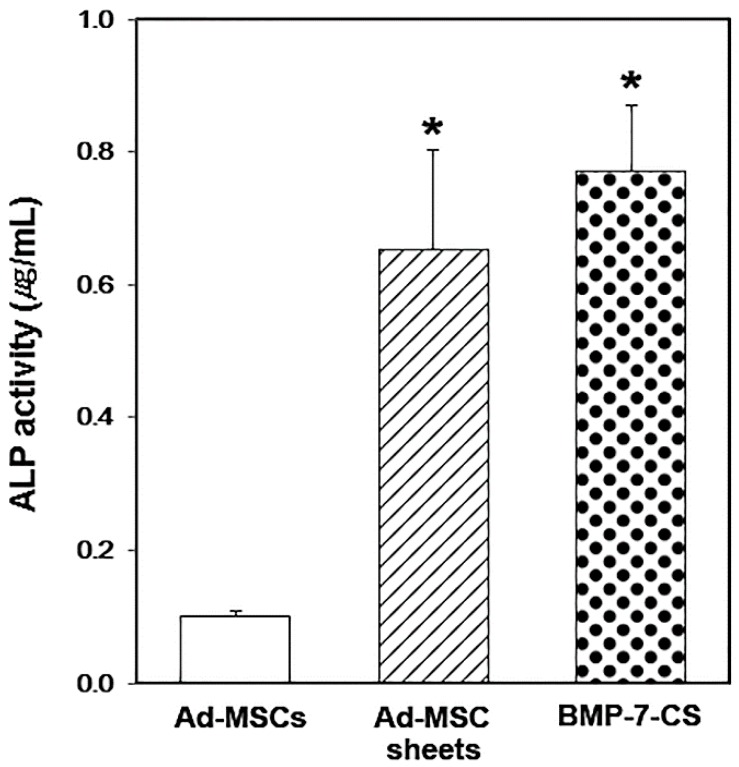
Quantification of alkaline phosphatase (ALP) activity. ALP activity was significantly higher in the Ad-MSC sheets and BMP-7 overexpressing Ad-MSC sheets (BMP-7-CS) than in the Ad-MSCs group (* *p* < 0.05).

The ALP activity of each group was assessed at 10 days after changing cell sheet medium. The ALP activity was enhanced 6.4- and 7.8-fold in Ad-MSC sheets and BMP-7-CS group, respectively compared to that of Ad-MSCs control group (*p* < 0.05, Figure 2).

### 2.3. Quantitative Reverse Transcription Polymerase Chain Reaction (qRT-PCR) Analysis 

**Figure 3:**
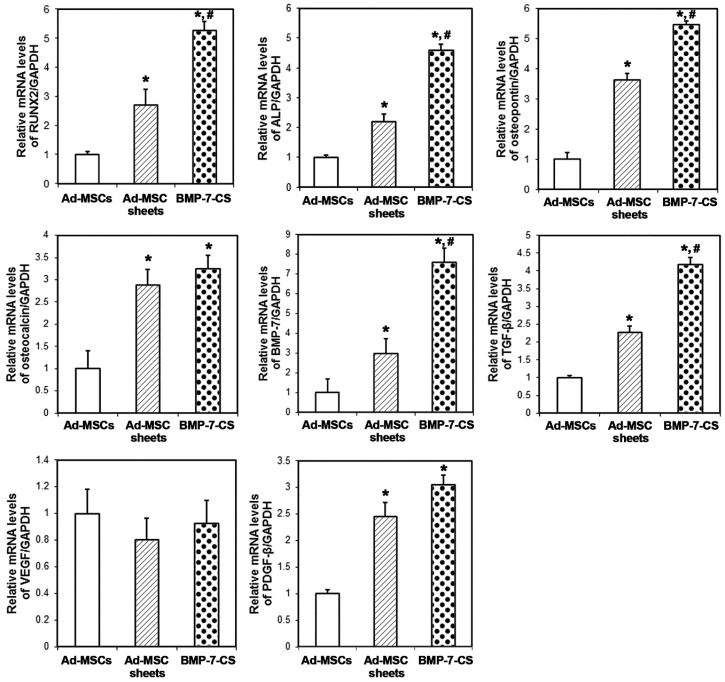
Expression of osteogenic differentiation and vascular-related markers. The expression of *runt-related transcription factor 2 (RUNX2), ALP*, *osteopontin, BMP7*, and *transforming growth factor (TGF)-β* mRNA was significantly upregulated in Ad-MSC sheets and BMP-7-CS (* *p* < 0.05). Except for *osteocalcin*, transcript levels of osteogenic differentiation markers were higher in BMP-7-CS than in the Ad-MSC sheets group (# *p* < 0.05). *PDGFB* mRNA expression was upregulated in Ad-MSC sheets and BMP-7-CS (* *p* < 0.05). *: Compared to the Ad-MSCs group, #: compared to the Ad-MSC sheets group.

The expression of *runt-related transcription factor 2 (RUNX2)*, *ALP*, *osteopontin*, *osteocalcin*, *BMP-7*, and *TGF-β* mRNA was significantly upregulated in Ad-MSC sheets and BMP-7-CS compared to that of Ad-MSCs control (*p* < 0.05, Figure 3). The transcript levels of almost all osteogenic differentiation markers were higher in the BMP-7-CS group than in the Ad-MSC sheets group (*p* < 0.05); the exception was the *osteocalcin*. Vascular-related markers were also investigated by evaluating *vascular endothelial growth factor (VEGF)* and *platelet-derived growth factor subunit B (PDGFB)* expression. The mRNA level of *VEGF* was not different among the groups. However, *PDGFB* mRNA was significantly upregulated in Ad-MSC sheets and BMP-7-CS compared to that of Ad-MSCs (*p* < 0.05).

### 2.4. In Vivo Bone Regeneration in Canine Radial Defects

**Figure 4:**
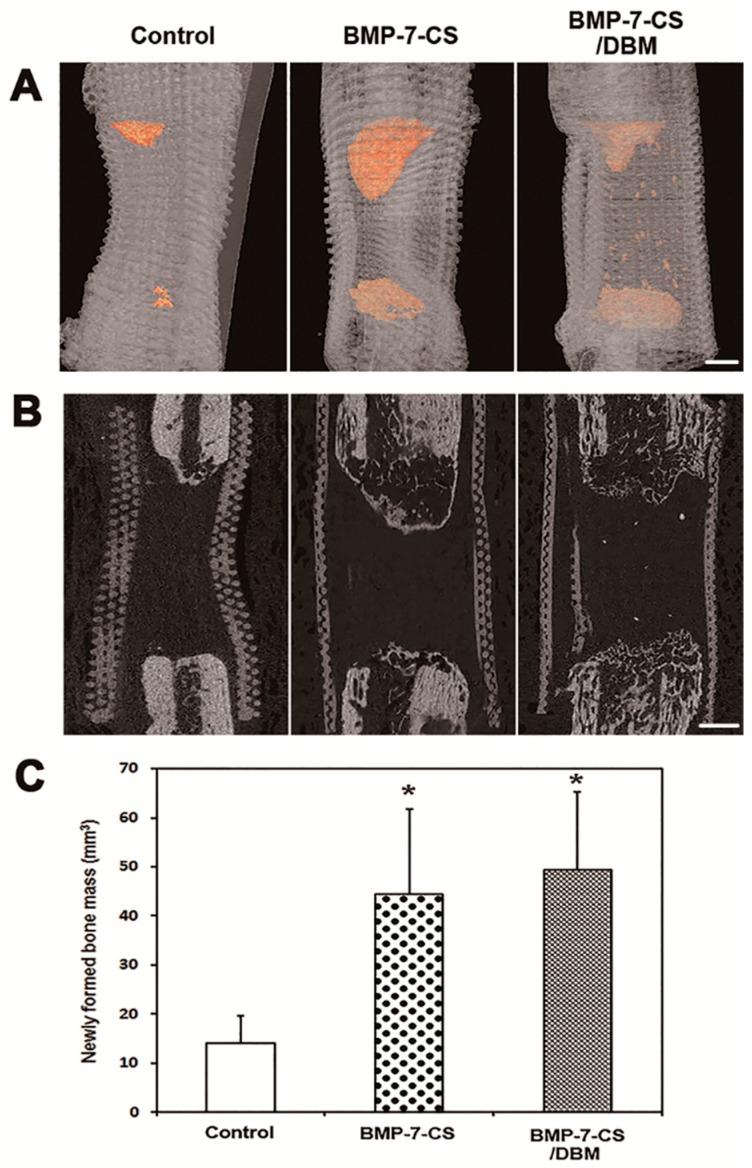
Bone regeneration in canine radial defects: (**A**) 3-D reconstructed image and (**B**) sagittal view image showed that new bone formation was detected within defects at the bone margin; (**C**) Quantitative 3-D micro-CT analysis revealed that groups with BMP-7-CS (with or without demineralized bone matrix (DBM)) showed a greater volume of newly formed bone than control groups (* *p* < 0.05). Moreover, mineralized bone particles were observed in the defect area of BMP-7-CS/DBM group. Bar: 3 mm.

New bone was detected within defects at each bone margin. In the three-dimensional (3-D) reconstructed image, the cone-shaped newly formed bone was visible (Figure 4A). From the sagittal view, the bone volume was discernible (Figure 4B), and a quantitative 3-D micro-computed tomography (CT) analysis revealed the following values for newly formed bone mass: control, 14.12 ± 5.54 cm^3^; BMP-7-CS, 44.37 ± 17.33 cm^3^; and BMP-7-CS/DBM, 49.30 ± 16.06 cm^3^ (Figure 4C). The amount of new bone formed was greater in BMP-7-CS and BMP-7-CS/DBM groups than in the control group (*p* < 0.05). Moreover, mineralized bone particles were observed in the defect area of the BMP-7-CS/DBM group.

### 2.5. Histological Evaluation

**Figure 5:**
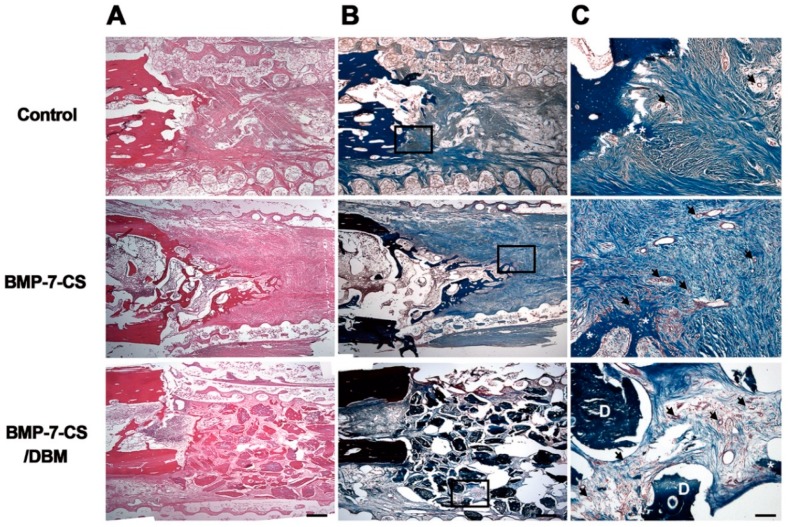
Histological analysis: (**A**) In hematoxylin and eosin staining, most of the defect areas were filled with fibrous connective tissue and newly formed bone tissue had a woven, trabecular appearance. Additionally, the defect site was filled with DBM particles in the BMP-7-CS/DBM group. (**B**,**C**) The Masson’s trichrome staining revealed abundant collagenous tissue around the regenerated tissue. Vasculature was observed around the new bone and DBM particles. Asterisks and arrows indicate bone tissue and vasculature, respectively. Bar: (**A**,**B**) 200 μm; (**C**) 25 μm.

**Figure 6:**
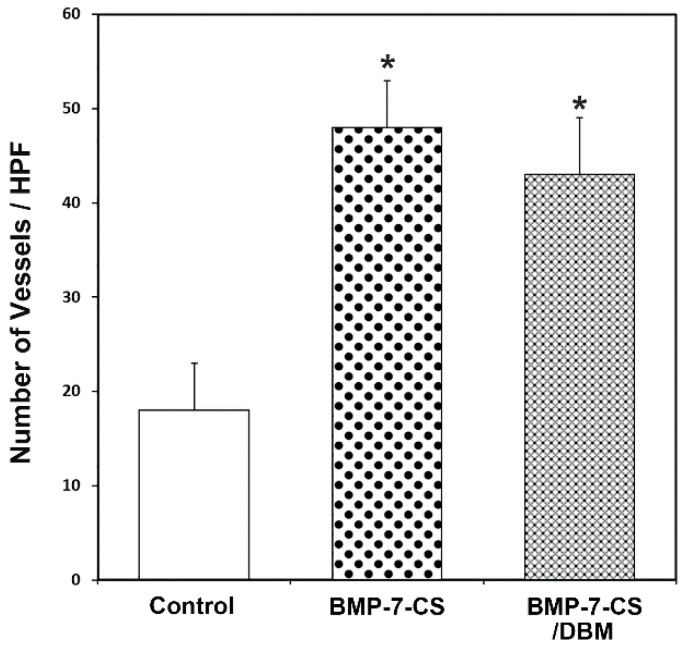
Neovascularization was determined by counting the number of vasculatures per high-power field. The BMP-7-CS and BMP-7-CS/DBM groups had significantly increased vasculature relative to the control group (* *p* < 0.05).

**Figure 7:**
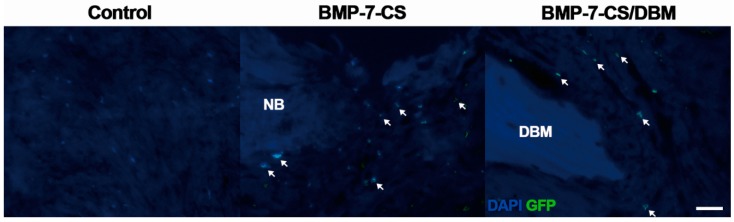
GFP expression at 12 weeks after transplantation of BMP-7-CS. In the BMP-7-CS and BMP-7-CS/DBM group, GFP labeled BMP-7-MSCs (white arrow) were observed around the new bone (NB) and DBM particles. 4′,6-diamidino-2-phenylindole (DAPI) stains were observed in the nucleus but GFP was not in the control. Bar: 25 μm.

At 12 weeks after implantation, decalcified paraffin sections were stained by hematoxylin and eosin and Masson’s trichrome to identify regenerated bone in defects. Newly formed bone was observed in longitudinal sections throughout the segmental defect of all groups (Figure 5A). In the control group, most of the defect areas were filled with fibrous connective tissue with minimal new bone formation. In the BMP-7-CS group, fibrous connective tissue was also observed and newly formed bone tissue had a woven, trabecular appearance. In the BMP-7-CS/DBM group, DBM particles mainly filled the defect and fibrous connective tissue existed among these particles as well as newly formed bone tissue also observed. Masson’s trichrome staining revealed abundant collagenous tissue around the regenerated tissue (Figure 5B). In addition, vasculature was observed around the new bone and DBM particles (Figure 5C). The BMP-7-CS and BMP-7-CS/DBM groups showed an increased number of vasculatures compared to that of control group (Figure 6). To determine the engraftment of BMP-7-MSCs, GFP was examined at 12 weeks after transplantation of BMP-7-CS. GFP labeled BMP-7-MSCs were observed around the new bone and DBM particles (Figure 7).

## 3. Discussion

The present study investigated the osteogenic potential of BMP-7-CS, as well as BMP-7-CS with and without DBM particles after transplantation into critical-sized bone defects in dogs. We previously conducted bone regeneration study in critical-sized bone defects model with cell sheets. The cell sheets showed greater osteogenic activities than MSCs transplantation. However, even though cell sheets were applied, the osteogenic outcomes were limited in the large bone defects [15]. To complement the results of our previous study, we designed the present study. First, we compared osteogenic potentials between AD-MSC sheets and BMP-7-CS. We chose the BMP-7-CS considering in vitro results. For maximized bone formation in the large bone defects, we applied BMP-7-CS and added DBM as osteoinductive and osteoconductive agent.

The scientific literature provides extensive evidence for the osteoblastic potential of MSCs [16]. An adequate supply of MSCs is important for efficient bone regeneration in large bone defects. However, injection of single-cell suspensions leads to uneven distribution and weak cell adhesion at target sites, which can ultimately result in cell death [17]. Cell sheet technology has been developed to enhance the regenerative capacity of tissue-engineered products [3,4]. Recently, a few techniques for creating cell sheets have been reported, such as thermo-responsive culture dishes grafted with a themoreponsive polymer, poly (N-isopropylacrylamine) or using l-ascorbic acid 2-phosphate (A2-P) [3,18,19]. The former method requires special culture dishes to create the cell sheets. In this study, a cell sheet created using A2-P, this method does not need any special equipment to create cell sheets. A2-P is a stable form of ascorbic acid that plays a role in collagen biosynthesis and ECM deposition [20]. For this methodology, sheets maintain intact cell-cell junctions and ECM that confer mechanical support to maintain the integrity of the transplant [21]. In addition, cell sheets delivered an abundance of cells compared with single-layered cells, because sheets were composed of two to four layers of cells [15]. 

Combination therapies with MSCs and growth factors were investigated to enhance bone repair. Of these growth factors, BMPs have been extensively studied as potent osteoinductive factors [22,23]. BMPs initiate the bone-healing cascade through the recruitment of MSCs from local bone and soft tissues and guide the differentiation of MSCs into osteoblasts. In the present study, we used a regional lentiviral gene delivery system, and successfully transduced the *BMP-7* gene into Ad-MSCs. BMP-7 MSCs secreted more BMP-7 than control Ad-MSCs. Regional gene therapy is a potential treatment option based on the sustained release of growth factors. Previous studies using lentiviral gene therapy demonstrated high levels of expression of BMP proteins for at least three months [8,24]. Similarly, in our study, transduced cells produced GFP at 12 weeks after transplantation. The longer duration of BMP production associated with the lentiviral vector was responsible for better bone repair in the large bone defects.

In the present study, BMP-7-CSs, which were produced from BMP-7-MSCs, showed strong osteogenic potential, as evidenced by upregulation of osteogenic differentiation markers such as *RUNX2*, *ALP*, *osteopontin*, and *osteocalcin* relative to Ad-MSCs and MSC-CSs. The ALP activity was also significantly higher in the Ad-MSC sheets and BMP-7-CS than in the Ad-MSCs group. However, it is not a revised value unlike the qRT-PCR, ALP activity was measured on a sheet or MSCs in a dish. As described above, sheet-formed MSCs such as Ad-MSC sheets and BMP-7-CS delivered more cells than Ad-MSCs. It seems to be increased ALP activity was more affected by cell number than BMP-7 overexpression. 

In addition, in our in vivo study, the BMP-7-CS group showed more extensive bone regeneration than the control group. During bone healing, the proliferation and osteoblastic differentiation of endogenous or exogenous MSCs are influenced by various growth factors, among which TGF-β and BMPs are important. BMP/TGF-β signaling induces MSC differentiation into osteoblasts via activation of intracellular pathways such as Smad and mitogen-activated protein kinase signaling [25,26]. TGF-β and BMPs transduce signals to both the canonical Smad-dependent signaling pathway and the non-canonical-Smad-independent signaling pathway to regulate MSCs differentiation during skeletal development, bone formation, and bone homeostasis [27]. Overexpression of BMP-7 from implanted MSCs can affect osteogenic differentiation of endogenous and exogenous MSCs. However, the donor cells appeared to function more as a BMP delivery vehicle than actually differentiating into osteoblasts [24]. In addition, it has been suggested that wrapped cell sheets function as a tissue-engineered periosteum at the defected bone segment. A biomimetic periosteum can maintain homeostasis of the cellular microenvironment by delivering growth factors.

The requirements for grafting material are substantial for regeneration in large bone defects. DBM is one such allograft material that has been shown to have osteoinductive and osteoconductive activities [28]. Early bone formation is related to the osteoconductive capacity of DBM, and allows osteoblast precursors to adhere to a collagen matrix similar to endogenous cortical bone matrix [29]. Although DBM by itself is believed to enhance bone formation, its osteogenic ability is not sufficient. The tissue-engineering approach is a promising strategy added in the field of bone regenerative medicine, with the goal of generating new cell-driven, functional tissues, rather than just implanting non-living scaffolds. In the present study, a high amount of new bone formation and calcium deposition inside the DBM particles was found in the BMP-7-CS/DBM group. Moreover, vascularization around newly formed bone and DBM particles was also identified. Neovascularization is necessary for bone regeneration. For this reason, the calcium deposited DBM is distinguished from partially decalcified DBM. 

Vascularization may have contributed to homeostasis of the microenvironment that promoted cells survival and bone formation. Some reports have proposed that MSCs and cell sheets stimulate bone formation by inducing vascularization [2,30]. Neovascularization alleviates hypoxia and is necessary for bone formation. VEGF and corresponding receptors are key regulators in a cascade of molecular and cellular events that ultimately lead to the development of the vascular system. In the present study, CSs and BMP-7-CSs expressed VEGF and PDGFB which corresponded to the formation of a vascular network around newly formed bone tissue following transplantation. Developed vascular beds around DBM particles might be involved in increased mineralization of DBM through osteoblast invasion. Neovascularization and the existence of several mineralized materials might enhance overall bone regeneration in critical size bone defects.

Gene therapy via gammaretroviruses, lentiviruses, and adenoviruses is attractive because of the natural ability of viruses to enter and deliver genetic material to cells. In the early days of gene therapy clinical trials, several side effects of the immune system had been reported [31,32]. However, third-generation lentiviral vectors improved safety by splitting the virial genome into separate plasmids. Moreover, introduction of deletion into the 3’ long terminal repeats of the viral genome creates self-inactivating lentiviral vectors, thus preventing unnecessary recombination [32,33,34]. Although there are theoretical potentials with lentiviral vectors, no cases have been reported. Additional research is still necessary to understand the long-term safety and efficacy of these vectors. 

## 4. Materials and Methods

### 4.1. Isolation and Culture of Canine Ad-MSCs 

The study protocol was approved by the Institutional Animal Care and Use Committee of Seoul National University (SNU-150624-7; 15 July 2015). MSCs derived from canine hip adipose tissue were isolated and characterized [35]. The tissue was collected aseptically from the subcutaneous fat of a 2-year-old beagle dog under anesthesia, and washed with Dulbecco’s phosphate-buffered saline (DPBS; Thermo Fisher Scientific Inc., Waltham, MA, USA), minced, and digested with collagenase type I (1 mg/mL; Sigma-Aldrich, St. Louis, MO, USA) at 37 °C for 30–60 min with intermittent shaking. The suspension was filtered through a 100 μm nylon mesh and centrifuged to separate floating adipocytes from stromal cells. Pre-adipocytes in the stromal vascular fraction were plated at 8000–10,000 cells/cm^2^ in T175 culture flasks containing Dulbecco’s modified Eagle’s medium (Thermo Fisher Scientific Inc.) supplemented with 3.7 g/L sodium bicarbonate, 1% penicillin/streptomycin, 1.7 mM L-glutamine, and 10% fetal bovine serum (Thermo Fisher Scientific Inc.). Cells were incubated in a humidified atmosphere at 37 °C and 5% CO_2_. Unattached cells and residual non-adherent red blood cells were removed after 24 h by washing with phosphate-buffered saline, and the culture medium was changed every 48 h. Cells were used for experiments after the third passage. 

### 4.2. Lentiviral Packing and Transduction

**Figure 8:**
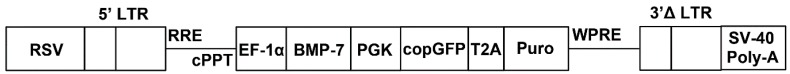
Construction of lentiviral vector. Lentiviral vectors contain an EF-1α promoter, BMP-7, copGFP, and puromycin genes. RSV: Rous sarcoma virus U3, LTR: long terminal repeat, RRE: Rev-responsible element, cPPT: central polyprine tract, WPRE: woodchuck hepatitis virus post-transcriptional regulatory element.

The canine *BMP-7* gene was cloned in reference to the gene database. A lentiviral vector encoding BMP-7 and GFP cDNA downstream of the elongation factor-1 alpha promoter was constructed (Figure 8). Twenty-four hours before transfection, 4 × 10^6^ HEK293T cells were seeded in a 100 mm dish. The following day, a lentiviral packaging mix (System Biosciences, San Diego, CA, USA) and lentiviral transgene plasmids were transfected into each well to create lentivirus. Virus particles were collected and transduced into Ad-MSCs at passage one. After Ad-MSCs reached 90% confluence, the stable cells were selected using puromycin (3 μg/mL, Thermo Fisher Scientific Inc.). Ad-MSCs were subcultured, and passage three cells were used for the following experiments. 

### 4.3. Gene Expression Analysis for Identification of BMP-7 Overexpression

Total RNA was isolated from Ad-MSCs or BMP-7-MSCs using the Hybrid-RTM RNA extraction kit (GeneAll Bio, Seoul, Korea) according to the manufacturer’s protocol. RNA concentration was determined by measuring optical density at 260 nm with a NanoDrop ND-1000 spectrophotometer (Nano Drop Technologies, Wilmington, DE, USA). cDNA was synthesized from RNA using a commercially available cDNA synthesis kit (Takara Bio, Otsu, Japan). qRT-PCR was performed with an ABI 7300 Real-time-PCR system (Applied Biosystems, Irvine, CA, USA), using SYBR Premix Ex Taq (Takara Bio). *BMP-7* primer sequences are listed in Table 1. Expression levels of target genes were normalized to the level of *glyceraldehyde 3-phosphate dehydrogenase (GAPDH)*, and quantitated with the ΔΔ*C*_t_ method [36].

### 4.4. Protein Expression Analysis for Identification of BMP-7 Overexpression

Ad-MSCs and BMP-7-MSCs were used for Western blot analysis. Briefly, the cells were washed twice with DPBS, and sonicated in lysis buffer (150 mM sodium chloride, 1% Triton X-100, 1% sodium deoxycholate, 0.1% sodium dodecyl sulfate (SDS), 50 mM Tris at pH 7.5, 2 mM ethylenediaminetetraacetic acid) on ice for 30 min. Lysates were cleared by centrifugation (10 min at 13,000× *g* and 4 °C), and protein concentrations were determined using the Bradford method [37]. Equal amounts of protein (15 μg) were resolved by electrophoresis on 10% SDS-polyacrylamide gels and transferred to polyvinylidene fluoride membranes. Membrane blots were washed with TBST (10 mM Tris-HCl, pH 7.6, 150 mM NaCl, 0.05% Tween-20), blocked with 5% skim milk for 1 h, and incubated with the appropriate primary antibodies at the recommended dilutions. The antibodies used included antibodies against actin (A3853, Sigma-Aldrich), BMP-7 (ab56023, Abcam, Cambridge, UK). The primary antibodies (1:1000) were diluted in TBST. The membrane was then washed, and primary antibodies were detected with goat anti-rabbit IgG or goat anti-mouse IgG conjugated to horseradish peroxidase (1:5000, Invitrogen, Carlsbad, CA, USA). Bands were visualized using enhanced chemiluminescence (Invitrogen). 

### 4.5. Preparation of Ad-MSC Sheets and BMP-7-CS

Ad-MSC sheets and BMP-7-CS were prepared as previously described [3]. Briefly, Ad-MSCs and BMP-7-MSCs were seeded at a density of 1 × 10^4^ cells/cm^2^ in a 100 mm culture dish and cultured in growth medium containing 82 μg/mL A2-P (Sigma-Aldrich) for 10 days. Ad-MSCs (negative control) were cultured in unsupplemented growth medium for 10 days.

### 4.6. Gene Expression Analysis

The expression of osteogenic differentiation markers and vascular-related markers were investigated on day 10. Total RNA was isolated from cells or cell sheets and qRT-PCR was performed as previously mentioned. Primer sequences are listed in Table 1. 

### 4.7. ALP Activity Measurement

Cells cultured in 100 mm dishes were used for the measurement of ALP activity using an ALP assay kit (Takara Bio) according to the manufacturer’s instructions. Briefly, p-nitrophenyl phosphate (pNPP) solution was prepared by dissolving 24 mg pNPP substrate in 5 mL ALP buffer. Cells were scraped into 200 μL of extraction solution, homogenized, and sonicated. The cleared supernatant was collected after centrifugation at 13,000× *g* at 4 °C for 10 min. A 50 μL volume of cell lysate supernatant was mixed with 50 μL of pNPP substrate solution and incubated at 37 °C for 30 min. After adding 50 μL of stop solution (0.5 N NaOH), absorbance was measured at 405 nm with a spectrophotometer. 

### 4.8. Fabrication of Poly ε-caprolactone/β-tricalcium phosphate Scaffolds

Poly ε-caprolactone (PCL) was dissolved in chloroform at 40 °C. NaCl and β-tricalcium phosphate (β-TCP) were ground and sieved, and granules between 25 and 33 μm were selected. β-TCP was prepared by calcination of nano-TCP (Merck, Kenilworth, NJ, USA) at 1000 °C for 4 h. Selected NaCl granules were mixed with predetermined amounts of ceramic particles (1:1 = NaCl:PCL, 1.5:1 = ceramic:PCL, weight ratios). Combined powders were mixed with the PCL suspension to produce a homogeneous paste. Sheet-type porous scaffolds (50 mm × 25 mm × 2 mm) were constructed by extruding the gel paste onto a substrate using a 3-D printing system. The shapes and sizes of the PCL/β-TCP scaffolds were designed using a computer system. NaCl was removed by immersing the scaffold in deionized water to produce macro-sized pores in strut and the water was replaced every 2 h with fresh water at 30 °C after sufficient drying of the scaffold. 

### 4.9. Animal Experiments

Beagle dogs (*n* = 12; 2–3 years old) weighing 9.1 ± 1.6 kg were used for this study. Dogs were handled in accordance with the animal care guidelines of the Institute of Laboratory Animal Resources, Seoul National University, Korea. The dogs were assigned to one of three groups (*n* = 4 for each group), including control, BMP-7-CS, and BMP-7-CS/DBM. For all groups, the defects were surrounded with PCL/β-TCP. For the BMP-7-CS group, PCL/β-TCP was wrapped with four BMP-7-CSs after 10 days of culture. Additionally, DBM particles (Veterinary tissue bank, Wrexham, UK) were placed in the defects for the BMP-7-CS/DBM group. The Institutional Animal Care and Use Committee of Seoul National University approved the experimental design. Dogs were medicated and anesthetized with tramadol (4 mg/kg by intravenous (i.v.) injection; Toranzin; Samsung Pharmaceutical Co., Hwasung, Korea), propofol (6 mg/kg i.v.; Provive; Claris Lifesciences, Ahmedabad, India), and atropine sulfate (0.05 mg/kg by subcutaneous injection; Jeil Pharmaceutical Co., Seoul, Korea). Anesthesia was maintained with isoflurane (Forane solution, Choongwae Pharmaceutical Co., Seoul, Korea) at a 1.5 minimum alveolar concentration throughout the procedure. Electrocardiography, pulse oximetry, respiratory gas analysis, and rectal temperature measurement were performed using an anesthetic monitoring system (Datex-Ohmeda S/5; GE Healthcare, Chicago, IL, USA). Under sterile conditions, a craniomedial incision was made in the skin to expose the diaphysis of the left radius. A 15 mm-long segmental defect was made in the middle portion of the diaphysis using an oscillating saw (Stryker, Kalamazoo, MI, USA) as previous described [38,39]. Overlying periosteum was also resected. Defects were surrounded by the experimental scaffold and filled with and without DBM. A nine-hole, 2.7 mm dynamic compression plate (Depuy Synthes, Dulliken, Switzerland) was placed on the cranial aspect of the radius. The soft tissue was closed with 3-0 polydioxanone sutures (Ethicon, Somerville, NJ, USA), and the skin was closed with 4-0 nylon sutures. All the animals were bandaged for 2 days after operation. Operated limbs were weight-beared after removing bandage.

### 4.10. Micro-CT for Bone Imaging

Dogs were sacrificed 12 weeks after implantation. The radius segment was excised, trimmed, and fixed in 10% formaldehyde. Samples were scanned using a micro-CT system (Skyscan; Bruker, Brussels, Belgium) and 3-D images were reconstructed; the volume of newly formed bone within bone defects was calculated using the auxiliary software (Bruker). 

### 4.11. Histological Analysis

After micro-CT measurements, specimens were decalcified in 8% nitric acid for 2 weeks at room temperature, dehydrated through a graded series of alcohol, embedded in paraffin, sectioned at a thickness of 5 μm, and stained with hematoxylin and eosin or Masson’s trichrome according to standard protocols. For the tracking of BMP-7-CS expressing GFP, the tissue was stained with 4′6-diamidino-2-phenylindole (DAPI, 1:100, Sigma-Aldrich) to identify nuclei and observed under a fluorescence microscope (Life technologies, Carlsbad, CA, USA). 

### 4.12. Statistical Analysis

Results are expressed as the mean ± standard deviation. Statistical analysis was performed using SPSS v.22.0 software (IBM, Armonk, NY, USA). Group means were compared using Kruskal–Wallis tests followed by Mann–Whitney *U* tests. A *p* value of less than 0.05 was considered statistically significant.

## 5. Conclusions

It was demonstrated that lentiviral-mediated gene transfer of BMP-7 into Ad-MSCs allows for stable production of BMP-7. The BMP-7-CSs also showed strong osteogenic potential. The properties of BMP-7 were well executed when the BMP-7 MSCs were delivered in cell sheets combined with DBM. The BMP-7-CSs might not only provide BMP-7 producing MSCs with ECM but also osteogenic and vascular trophic factors which when combined with the osteoinduction and osteoconduction properties of DBM, result in synergy during bone regeneration. Our findings indicate that lentiviral gene therapy can offer prolonged gene expression and could be ideal for the treatment of large bone defects that require a long-term osteoinductive stimulus. In addition, cooperation between BMP-7-CSs and DBM has therapeutic potential for the treatment of critical-sized bone defects and could be used to enhance current treatment practices.

## Figures and Tables

**Table 1:** Primers sequences used for quantitative reverse transcription PCR.

Target Gene	Gene Bank Access Number	Primers Sequence (5′–3’)		bp
*RUNX2*	XM_022425793.1	Forward	TGTCATGGCGGGTAACGAT	107
		Reverse	TCCGGCCCACAAATCTCA	
*ALP*	AF540075.1	Forward	TCCGAGATGGTGGAAATAGC	272
		Reverse	GGGCCAGACCAAAGATAGAG	
*Osteopontin*	DQ195101.1	Forward	GATGATGGAGACGATGTGGATA	116
		Reverse	TGGAATGTCAGTGGGAAAATC	
*Osteocalcin*	XM_014115322.2	Forward	CTGGTCCAGCAGATGCAAAG	207
		Reverse	GGTCAGCCAGCTCGTCACAGTT	
*BMP-7*	NM_001197052.1	Forward	TCGTGGAGCATGACAAAGAG	135
		Reverse	GCTCCCGAATGTAGTCCTTG	
*TGF-β*	NM_001003309.1	Forward	CTCAGTGCCCACTGTTCCTG	215
		Reverse	TCCGTGGAGCTGAAGCAGTA	
*VEGF*	NM_001003175.2	Forward	CTATGGCAGGAGGAGAGCAC	288
		Reverse	GCTGCAGGAAACTCATCTCC	
*PDGFB*	NM_001003383.1	Forward	CCGAGGAGCTCTACGAGATG	150
		Reverse	AACTCTCCAGCTCGTCTCCA	
*GAPDH*	NM_001003142.2	Forward	CATTGCCCTCAATGACCACT	105
		Reverse	TCCTTGGAGGCCATGTAGAC	

## Abbreviations

Ad-MSCs	Adipose-derived mesenchymal stem cells
ALP	Alkaline phosphatase
BMP-7-CS	Bone morphogenetic proteins overexpressing adipose-derived mesenchymal stem cell sheets
BMP	Bone morphogenetic proteins
DBM	Demineralized bone matrix
ECM	Endogenous extracellular matrix
A2-P	l-Ascorbic acid 2-phosphate
MSC	Mesenchymal stem cells
PDGFB	Platelet-derived growth factor subunit B
PCL	Poly ε-caprolactone
RUNX2	Runt-related transcription factor 2
TGF	Transforming growth factor
VEGF	Vascular endothelial growth factor
β-TCP	β-Tricalcium phosphate
